# Ischemic injury leads to extracellular matrix alterations in retina and optic nerve

**DOI:** 10.1038/srep43470

**Published:** 2017-03-06

**Authors:** Jacqueline Reinhard, Marina Renner, Susanne Wiemann, Daniel A. Shakoor, Gesa Stute, H. Burkhard Dick, Andreas Faissner, Stephanie C. Joachim

**Affiliations:** 1 Department of Cell Morphology and Molecular Neurobiology, Faculty of Biology and Biotechnology, Ruhr-University Bochum, Universitätsstrasse 150, 44780 Bochum, Germany; 2 Experimental Eye Research Institute, University Eye Hospital, Ruhr-University Bochum, In der Schornau 23-25, 44892 Bochum, Germany

## Abstract

Retinal ischemia occurs in a variety of eye diseases. Restrained blood flow induces retinal damage, which leads to progressive optic nerve degeneration and vision loss. Previous studies indicate that extracellular matrix (ECM) constituents play an important role in complex tissues, such as retina and optic nerve. They have great impact on de- and regeneration processes and represent major candidates of central nervous system glial scar formation. Nevertheless, the importance of the ECM during ischemic retina and optic nerve neurodegeneration is not fully understood yet. In this study, we analyzed remodeling of the extracellular glycoproteins fibronectin, laminin, tenascin-C and tenascin-R and the chondroitin sulfate proteoglycans (CSPGs) aggrecan, brevican and phosphacan/RPTPβ/ζ in retinae and optic nerves of an ischemia/reperfusion rat model via quantitative real-time PCR, immunohistochemistry and Western blot. A variety of ECM constituents were dysregulated in the retina and optic nerve after ischemia. Regarding fibronectin, significantly elevated mRNA and protein levels were observed in the retina following ischemia, while laminin and tenascin-C showed enhanced immunoreactivity in the optic nerve after ischemia. Interestingly, CSPGs displayed significantly increased expression levels in the optic nerve. Our study demonstrates a dynamic expression of ECM molecules following retinal ischemia, which strengthens their regulatory role during neurodegeneration.

Retinal ischemia is defined as chronically restrained blood flow to the eye. There are many causes of retinal ischemia, including age-dependent macular degeneration, central vein occlusion, diabetic retinopathy or glaucoma^1^,^2^,^3^. Due to the restrained blood flow, retinal ischemia is accompanied by a loss of nutrient and oxygen supply, oxidative stress or increased glutamatergic stimulation and results in severe neuronal damage as well as impaired retinal function^4^,^5^.

Although immediate reperfusion limits retinal damage, an excessive generation of reactive oxygen species and inflammatory processes accelerate neuronal loss and death^6^,^7^,^8^. However, in comparison to the brain, the retina exhibits a certain resistance to ischemic injury, which might also depend on the unique retinal microenvironment^9^. Nevertheless, due to the inhibitory environment neuronal/axonal regeneration capacity is severely limited in the mature retina^10^,^11^,^12^. Previous studies indicate that extracellular matrix (ECM) components play an important functional role in the developing and diseased retina. These ECM constituents form a network of glycoproteins and proteoglycans, provide mechanical and structural support and regulate cellular homeostasis as well as signaling. Moreover, ECM molecules display a great impact on de- and regeneration processes besides representing major candidates of central nervous system (CNS) glial scar formation or during retinal degeneration^13^. For instance, especially proteoglycans have been described to exhibit a protective influence on retinal ganglion cells (RGCs)^14^,^15^,^16^. Inatani *et al*. reported an upregulation of the chondroitin sulfate proteoglycan (CSPG) neurocan following transient retinal ischemia^17^. In contrast, decorin, a CSPG and dermatan sulfate proteoglycan (DSPG), was described to be transiently downregulated after retinal ischemia^18^. Nevertheless, the importance of ECM constituents during ischemic retinal neurodegeneration is not fully understood yet and so far, little information exists regarding the expression pattern of the ECM in the ischemic retina as well as optic nerve.

The retinal ischemia/reperfusion (I/R) rat model represents an excellent model to investigate the consequences of retinal damage. In this study, rats underwent 60 min of retinal ischemia in one eye, followed by reperfusion^19^. In our study, we analyzed the remodeling of the ECM glycoproteins fibronectin, laminin, tenascin-C and tenascin-R and the CSPGs aggrecan, brevican and phosphacan/RPTPβ/ζ (receptor protein tyrosine phosphatase β/ζ) in retinae and optic nerves of an I/R model. The expression pattern of the ECM molecules was evaluated on mRNA level via quantitative real-time PCR (qRT-PCR) and on protein level through Western blot quantification and immunohistochemistry.

## Results

### ECM glycoproteins in the control and ischemic retina and optic nerve

**Figure 1:**
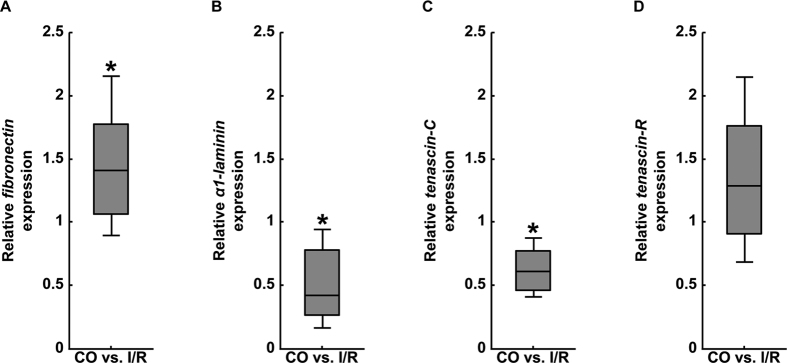
Analyses of relative *fibronectin* (**A**), *α1-laminin* (**B**), *tenascin-C* (**C**) and *tenascin-R* (**D**) mRNA expression using qRT-PCR in control (CO) and ischemic (I/R) retinae. Our results revealed significantly elevated levels for the glycoprotein *fibronectin* in I/R retinae, whereas *α1-laminin* and *tenascin-C* displayed a significantly reduced expression. No expression changes were observed for *tenascin-R*. Values are median ± quartile ± maximum/minimum. *p ≤ 0.05; n = 4/group.

First, we analyzed the expression pattern of the ECM glycoproteins fibronectin, *α1-*laminin, tenascin-C and -R in control (CO) and ischemic (I/R) retinae via qRT-PCR (Fig. 1A–D). Regarding the mRNA expression pattern of the aforementioned glycoproteins, we observed a significant upregulation of *fibronectin* in the I/R group (1.41-fold, p = 0.032; Fig. 1A). In contrast, both *α1-laminin* (0.42-fold, p = 0.031; Fig. 1B) as well as *tenascin-C* (0.61-fold, p = 0.027; Fig. 1C) mRNA levels were significantly downregulated in I/R retinae in comparison to CO tissue. For *tenascin-R* no significant difference was observed between both groups (1.29-fold, p = 0.219; Fig. 1D).

**Figure 2:**
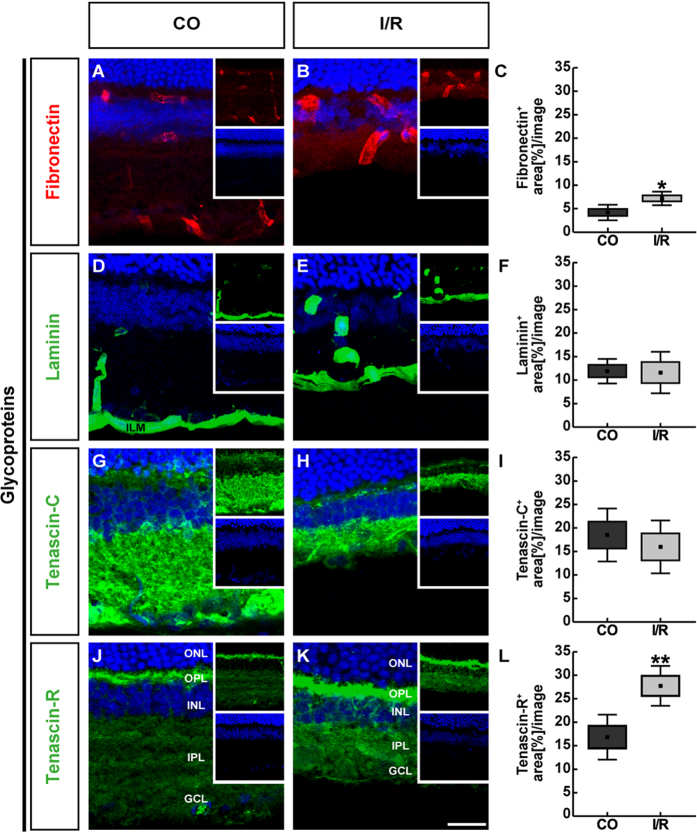
Representative retinal cross-sections of control (CO) and ischemic (I/R) eyes stained using specific antibodies directed against the ECM glycoproteins fibronectin (**A**,**B**, red), laminin (**D**,**E**, green), tenascin-C (**G**,**H**, green) and tenascin-R (**J**,**K**, green) as well as the nuclear dye TO-PRO-3 (blue) shown as merged images. Small inserts display TO-PRO-3 (blue) and glycoprotein (green/red) staining separately. In both experimental groups, fibronectin as well as laminin displayed a blood vessel-associated staining. In addition, laminin was found in the ILM and in the GCL. Tenascin-C and -R staining was mainly localized in the IPL, OPL and GCL. Quantification revealed a significant increase in the fibronectin and tenascin-R staining area in ischemic retinae, whereas no significant changes were observed regarding the tenascin-C and laminin staining (**C**,**F**,**I**,**L**). Values are mean ± SEM ± SD. *p ≤ 0.05; **p ≤ 0.01; n = 5/group. GCL = ganglion cell layer, ILM = inner limiting membrane, INL = inner nuclear layer, IPL = inner plexiform layer, ONL = outer nuclear layer, OPL = outer plexiform layer. Scale bar = 50 μm.

**Figure 3:**
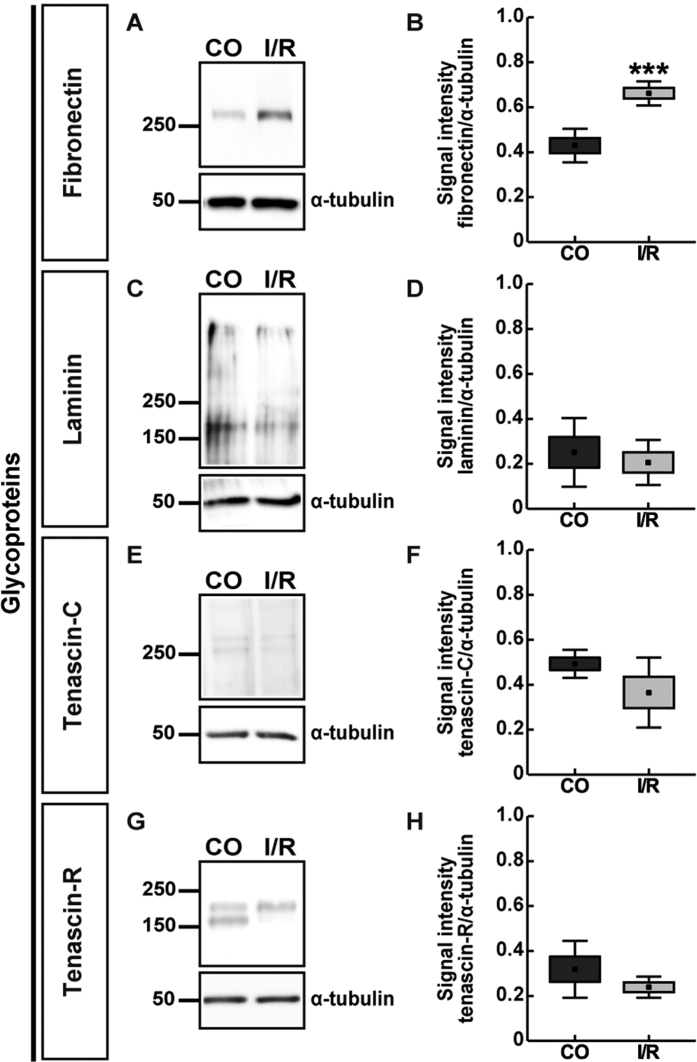
Western blot analyses of relative fibronectin (**A**,**B**), laminin (**C**,**D**), tenascin-C (**E**,**F**) and tenascin-R (**G**,**H**) protein levels in control (CO) and ischemic (I/R) retinae. Relative protein quantification revealed a significant upregulation of fibronectin levels. Comparable protein levels were observed for laminin, tenascin-C and tenascin-R in the ischemic and control retinae. Values are indicated as mean ± SEM ± SD. ***p < 0.001, n = 5/group.

To further analyze the distribution pattern of these glycoproteins, we labeled horizontal retinal sections with specific antibodies and analyzed their immunoreactivity by semi-quantitative area analyses (Fig. 2A–L). Regarding fibronectin staining, specific signals were restricted to retinal blood vessels of control and ischemic retinae (Fig. 2A,B). Analyses of fibronectin immunoreactivity revealed a significant area increase in ischemic (7.19 ± 1.45 area [%]/image; p = 0.016) in comparison to CO retinae (4.19 ± 1.64 area [%]/image; Fig. 2C). By Western blot analyses of retinal cell lysates, fibronectin was observed at >250 kDa (Fig. 3A,B). In line with the qRT-PCR and immunohistochemical results, quantitative protein analyses verified a significantly increased protein level in I/R (0.66 ± 0.05; p < 0.001) compared to CO retinae (0.43 ± 0.07).

For the glycoprotein laminin, we observed a prominent immunoreactivity in retinal blood vessels, the inner limiting membrane (ILM) as well as in close association with RGCs within CO and I/R retinae (Fig. 2D,E). Here, our analyses showed no significant differences in immunoreactivity of ischemic (11.59 ± 5.04 area [%]/image; p = 0.906) and CO retinae (11.91 ± 2.98 area [%]/image; Fig. 2F). Also, protein levels of laminin, detected at 200 and 400 kDa via quantitative Western blot analyses, were comparable in I/R (0.21 ± 0.10; p = 0.601) and CO retinae (0.25 ± 0.15; Fig. 3C,D).

In both groups, tenascin-C immunoreactivity was mainly localized to the inner plexiform layer (IPL) and outer plexiform layer (OPL). Additionally, tenascin-C signals were seen in the inner nuclear layer (INL) and the ganglion cell layer (GCL) (Fig. 2G,H). Analyses of the tenascin-C staining area revealed no significant difference in I/R (15.98 ± 6.43 area [%]/image; p = 0.552) compared to CO (18.50 ± 6.42 area [%]/image; Fig. 2I) retinae. Western blot analyses of total retinal lysates also revealed comparable band intensities of tenascin-C protein levels (CO: 0.49 ± 0.06; I/R: 0.37 ± 0.16; p = 0.127; Fig. 3E,F). Nevertheless, a significant downregulation of the 250 kDa band was observed (CO: 0.51 ± 0.08; I/R: 0.35 ± 0.13; p = 0.04; data not shown).

Tenascin-R immunoreactivity was specifically enriched in the OPL, INL, IPL and GCL (Fig. 2J,K). Here, our immunofluorescence analyses showed a significant upregulation of tenascin-R area in ischemic (27.74 ± 4.85 area [%]/image; p = 0.01) compared to CO retinae (16.83 ± 5.44 area [%]/image; Fig. 2L). By Western blotting, tenascin-R was detected as two major bands at 160 and 180 kDa (Fig. 3G,H). Here, densitometric measurements of total tenascin-R protein revealed comparable levels (CO: 0.32 ± 0.13; I/R: 0.24 ± 0.05; p = 0.223). Interestingly, a significant upregulation of the larger tenascin-R isoform was observed in I/R (0.44 ± 0.09; p = 0.007; data not shown) compared to CO retinae (0.22 ± 0.10). In contrast, reduced protein levels were observed for the low molecular weight isoform in both groups (CO: 0.41 ± 0.15; I/R: 0.05 ± 0.02; p < 0.001; data not shown), indicating an isoform-specific regulation of tenascin-R.

**Figure 4:**
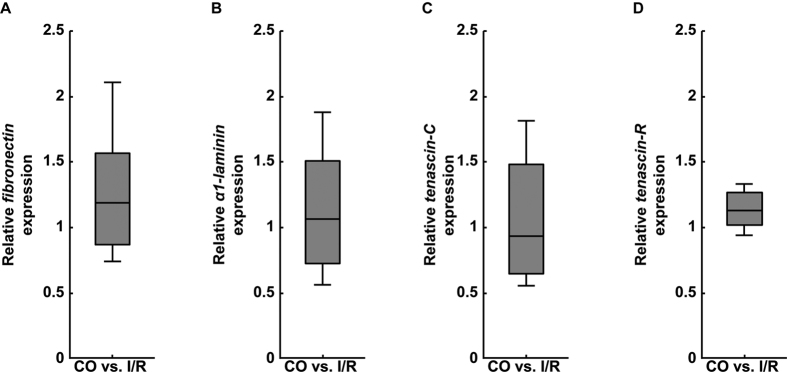
Analyses of relative *fibronectin* (**A**), *α1-laminin* (**B**), *tenascin-C* (**C**) and *tenascin-R* (**D**) mRNA expression using qRT-PCR in control (CO) and ischemic (I/R) optic nerves. In comparison to the CO group, no significant dysregulation was found for the investigated glycoproteins in ischemic optic nerves. Values are median ± quartile ± maximum/minimum; n = 4/group.

We also investigated the expression levels of *fibronectin, α1-laminin, tenascin-C* and *tenascin-R* in the CO and ischemic optic nerves by qRT-PCR analyses (Fig. 4A–D). On mRNA level, no significant regulation was observed for the investigated glycoproteins in I/R nerves (*fibronectin*: 1.19-fold, p = 0.352; *α1-laminin*: 1.07-fold, p = 0.826; *tenascin-C*: 0.93-fold, p = 0.75 and *tenascin-R:* 1.13-fold, p = 0.091; Fig. 4A–D).

**Figure 5:**
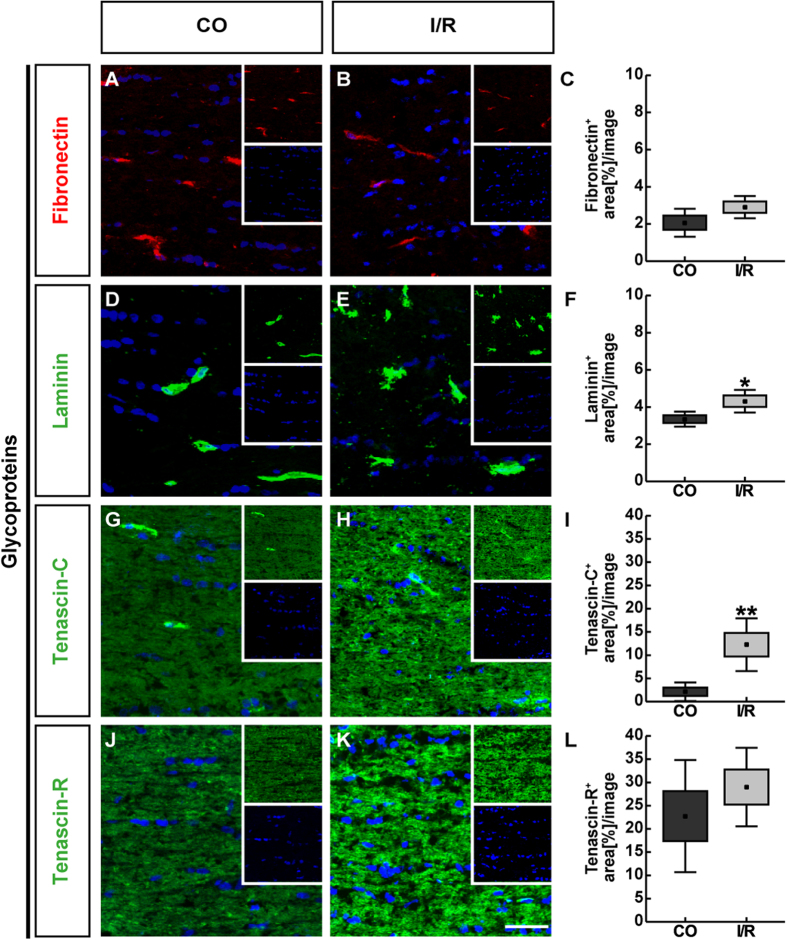
Representative longitudinal optic nerve sections of control (CO) and ischemic (I/R) eyes stained using specific antibodies directed against the ECM glycoproteins fibronectin (**A**,**B**, red), laminin (**D**,**E**, green), tenascin-C (**G**,**H**, green) and tenascin-R (**J**,**K**, green) as well as the nuclear dye TO-PRO-3 (blue) shown as merged images. Small inserts display TO-PRO-3 (blue) and glycoprotein (green/red) staining separately. Fibronectin as well as laminin staining was restricted to single cells within the optic nerve. In contrast, both tenascin proteins showed an extracellular staining pattern throughout the optic nerve. Analyses of the staining area verified a significant increase of laminin and tenascin-C in ischemic optic nerves. No significant increase was observed for fibronectin and tenascin-R (**C**,**F**,**I**,**L**). Values are mean ± SEM ± SD. *p ≤ 0.05; **p ≤ 0.01; n = 5/group. Scale bar = 50 μm.

As revealed by immunohistochemistry, fibronectin and laminin showed a distinct cellular expression pattern (Fig. 5A,B,D,E). Especially in the ischemic condition, both tenascins displayed a more widely extracellular staining pattern in optic nerve tissue (Fig. 5G,H,J,K). In line with qRT-PCR results, area analyses revealed no significant changes for fibronectin (CO: 2.07 ± 0.86 area [%]/image; I/R: 2.91 ± 0.69 area [%]/image; p = 0.126; Fig. 5A–C) and tenascin-R (CO: 22.75 ± 12.05 area [%]/image; I/R: 29.00 ± 8.45 area [%]/image; p = 0.371; Fig. 5J–L) via immunohistochemistry. A significant staining area increase was demonstrated for the glycoprotein laminin (CO: 3.35 ± 0.46 area [%]/image; I/R: 4.31 ± 0.70 area [%]/image; p = 0.032; Fig. 5D–F). Also, for tenascin-C a significantly increased area was observed in the ischemic group (12.24 ± 5.69 area [%]/image; p = 0.006) compared to controls (2.12 ± 2.01 area [%]/image; Fig. 5G–I).

### CSPGs in the control and ischemic retina and optic nerve

**Figure 6:**
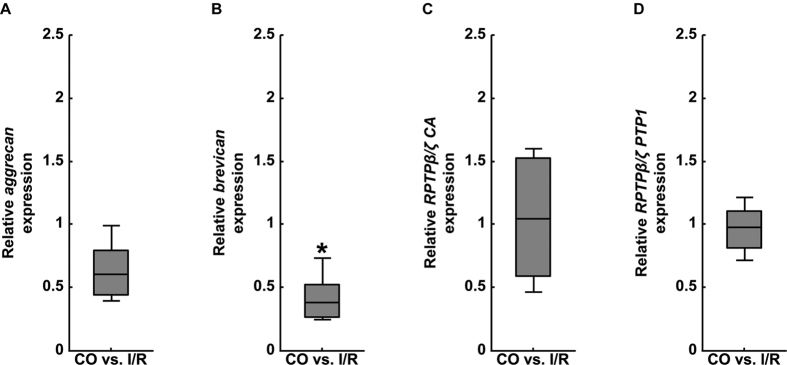
Analyses of relative *aggrecan* (**A**), *brevican* (**B**) and *phosphacan*/*RPTPβ/ζ* (RPTPβ/ζ CA and RPTPβ/ζ PTP1 primer pairs, **C**,**D**) mRNA expression using qRT-PCR in the retinae of control (CO) and ischemic (I/R) eyes. As indicated, significantly reduced expression levels were observed for the CSPG *brevican* in ischemic retinae. In contrast, no significant regulation was found for *aggrecan* and *phosphacan/RPTPβ/ζ*. Values are median ± quartile ± maximum/minimum. *p ≤ 0.05; n = 4/group.

Next, we analyzed expression levels of the CSPGs *aggrecan, brevican* and *phosphacan/RPTPβ/ζ* via qRT-PCR in CO and I/R retinae (Fig. 6A–D). Based on these analyses, we verified a significant downregulation of *brevican* expression levels (0.38-fold, p = 0.03; Fig. 6B). In contrast, comparable mRNA expression levels were seen for *aggrecan* (0.60-fold, p = 0.059; Fig. 6A), all *RPTPβ/ζ*-isoforms (*RPTPβ/ζ CA:* 1.04-fold, p = 0.884; Fig. 6C) as well as for the *RPTPβ/ζ* receptor variants (*RPTPβ/ζ PTP1:* 0.98-fold, p = 0.883; Fig. 6D) in both groups.

**Figure 7:**
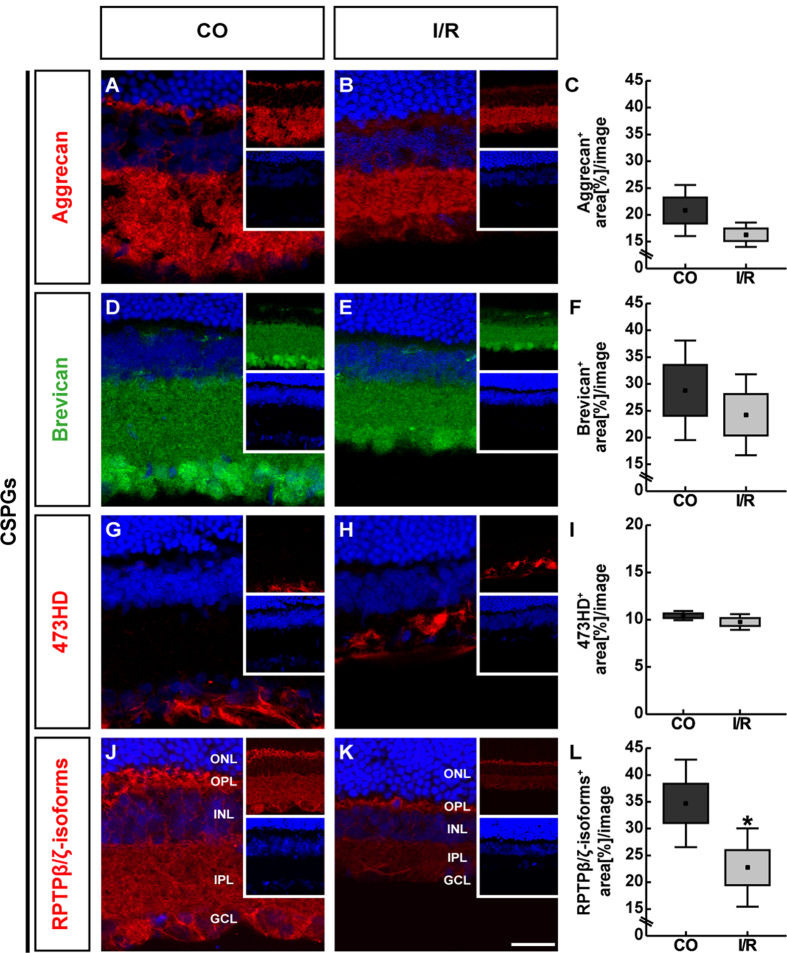
Representative retinal sections of control (CO) and ischemic (I/R) eyes stained using specific antibodies against the CSPGs aggrecan (**A**,**B**, red), brevican (**D**,**E**, green), phosphacan/RPTPβ/ζ_long_ (473HD antibody, **G**,**H**, red), RPTPβ/ζ-isoforms (**J**,**K**, red) as well as the nuclear dye TO-PRO-3 (blue) shown as merged images. Small inserts show TO-PRO-3 (blue) and CSPG (green/red) staining separately. For aggrecan and brevican as well as phosphacan/RPTPβ/ζ prominent immunoreactivity was found in the IPL and GCL. Brevican was also associated with cell somata of the GCL. 473HD immunoreactivity was restricted to Müller glia fibers and their endfeet, which form the ILM. Additionally, phosphacan/RPTPβ/ζ immunostaining was detectable in the OPL. The analyses revealed significantly less staining area in I/R retinae. No significant alteration was observed for aggrecan, brevican and 473HD immunoreactivity (**C**,**F**,**I**,**L**). Values are mean ± SEM ± SD. *p ≤ 0.05; n = 5/group. GCL = ganglion cell layer, INL = inner nuclear layer, IPL = inner plexiform layer, ONL = outer nuclear layer, OPL = outer plexiform layer. Scale bar = 50 μm.

**Figure 8:**
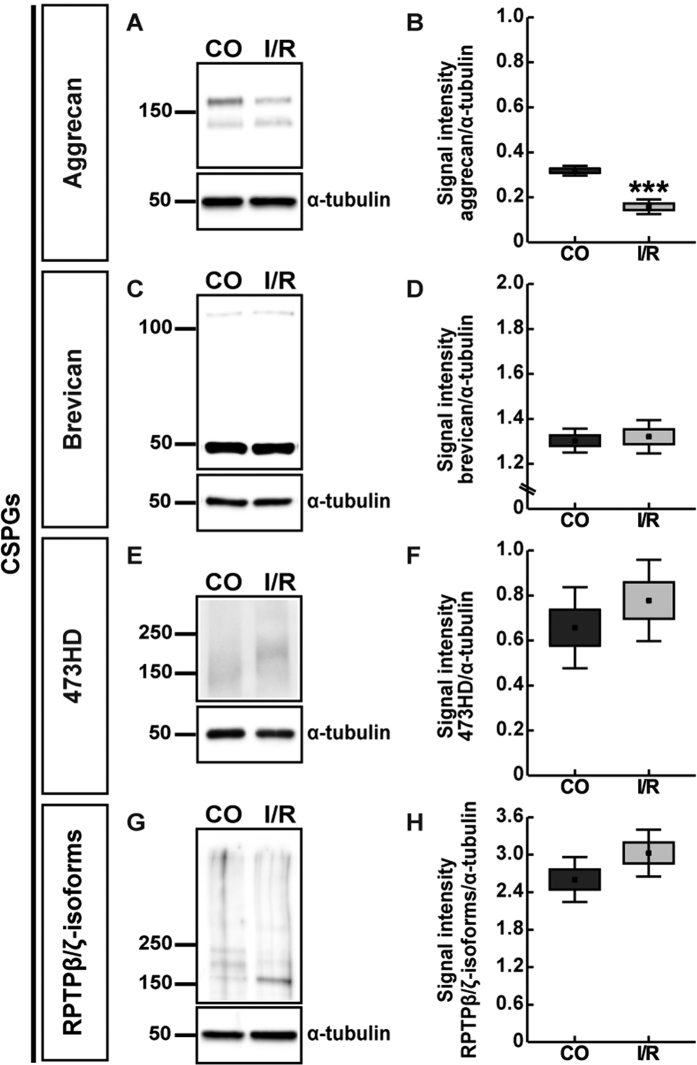
Western blot analyses of aggrecan (**A**,**B**), brevican (**C**,**D**), phosphacan/RPTPβ/ζ_long_ (473HD antibody, **E**,**F**) and RPTPβ/ζ-isoforms (**G**,**H**) protein levels in control (CO) and ischemic (I/R) retinae. Relative protein quantification revealed a significant downregulation of aggrecan levels. Comparable total protein levels were observed for brevican and RPTPβ/ζ-isoforms in ischemic and control retinae. Values are indicated as mean ± SEM ± SD. ***p ≤ 0.001, n = 5/group.

Further, we evaluated the immunohistochemical staining pattern of the CSPGs aggrecan, brevican and phosphacan/RPTPβ/ζ in the CO and I/R retinae (Fig. 7A-L). Concerning aggrecan and brevican, prominent immunoreactivity was localized in the IPL of the retinae (Fig. 7A,B,D,E). In addition, brevican immunostaining was prominently seen in the GCL, whereas aggrecan was also found in the IPL. Our statistical analyses revealed no significant changes for aggrecan (CO: 20.80 ± 5.44 area [%]/image; I/R: 16.28 ± 2.59 area [%]/image; p = 0.132; Fig. 7C) and brevican (CO: 28.80 ± 10.60 area [%]/image; I/R: 24.24 ± 8.62 area [%]/image; p = 0.477; Fig. 7F) staining area within the I/R group in comparison to the CO group. In contrast, quantitative analyses of total aggrecan protein, detected as two bands at >100 and >150 kDa, revealed a significantly reduced level in the I/R group compared to CO retinae (CO: 0.32 ± 0.02; I/R: 0.16 ± 0.03; p < 0.001; Fig. 8A,B). For brevican, prominent protein bands were detected at ~50 and >100 kDa (Fig. 8C,D). Here, relative quantification verified comparable total protein levels in I/R and CO retinae (CO: 1.30 ± 0.05; I/R: 1.32 ± 0.07; p = 0.683; Fig. 8C,D).

Next, we used an antibody against the 473HD epitope, a particular chondroitin sulfate glycan, specifically localized on the secreted splice variant phosphacan as well as RPTPβ/ζ_long_^20^,^21^. As demonstrated by previous studies and in this study, 473HD immunoreactivity is restricted to Müller glia cells (Fig. 7G,H)^13^,^22^,^23^. Due to the downregulation of the RPTPβ/ζ_long_ isoform within the adult retina, 473HD immunoreactivity mainly reflects the expression of the secreted phosphacan isoform. Analyses of the 473HD staining area showed no significant alteration between both groups (CO: 10.43 ± 0.55 area [%]/image; I/R: 9.76 ± 0.94 area [%]/image; p = 0.206; Fig. 7I), which is in line with the observed comparable mRNA levels revealed by qRT-PCR analyses. Western blotting using the 473HD antibody revealed a protein expression at >150 kDa. Quantitative analyses revealed comparable levels in CO and I/R retinae (CO: 0.66 ± 0.18; I/R: 0.78 ± 0.18; p = 0.32; Fig. 8E,F).

In addition, we used a polyclonal antibody, which is directed against all known three RPTPβ/ζ-isoforms, namely the secreted splice variant phosphacan and the receptor-types RPTPβ/ζ_long_ and RPTPβ/ζ_short_^20^. I/R retinae exhibited a reduced positive staining area for phosphacan/RPTPβ/ζ (Fig. 7J,K). A diffuse staining pattern was localized in the OPL, INL, IPL and GCL. As revealed by area analyses, a significantly reduced staining area was detected within I/R (22.74 ± 7.30 area [%]/image; p = 0.04) in comparison to CO retinae (34.71 ± 8.15 area [%]/image; Fig. 7L). By Western blot analyses, phosphacan/RPTPβ/ζ-isoforms were detected between 150 and >250 kDa. Here, relative quantification revealed comparable total protein levels in I/R and CO retinae (CO: 2.60 ± 0.36; I/R: 3.03 ± 0.38; p = 0.11; Fig. 8G,H). Strikingly, following ischemia the intermediate of three bands was significantly downregulated (CO: 0.21 ± 0.07; I/R: 0.09 ± 0.04; p = 0.011; data not shown).

**Figure 9:**
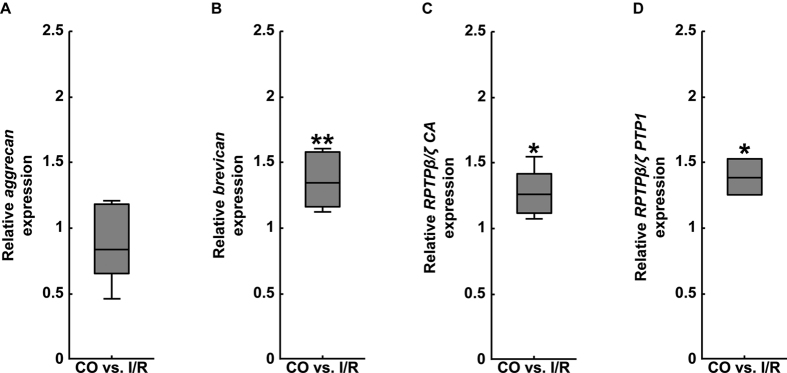
Analyses of relative *aggrecan* (**A**), *brevican* (**B**) and *phosphacan/RPTPβ/ζ* (RPTPβ/ζ CA and RPTPβ/ζ PTP1 primer pairs, **C**,**D**) mRNA expression using qRT-PCR in optic nerves of control (CO) and ischemic (I/R) eyes. Our expression analysis revealed a significant upregulation of *brevican* and *phosphacan/RPTPβ/ζ* in the optic nerve following ischemia. A comparable mRNA expression level was observed for *aggrecan* in both groups. Values are median ± quartile ± maximum/minimum. *p ≤ 0.05; **p ≤ 0.01; n = 4/group.

**Figure 10:**
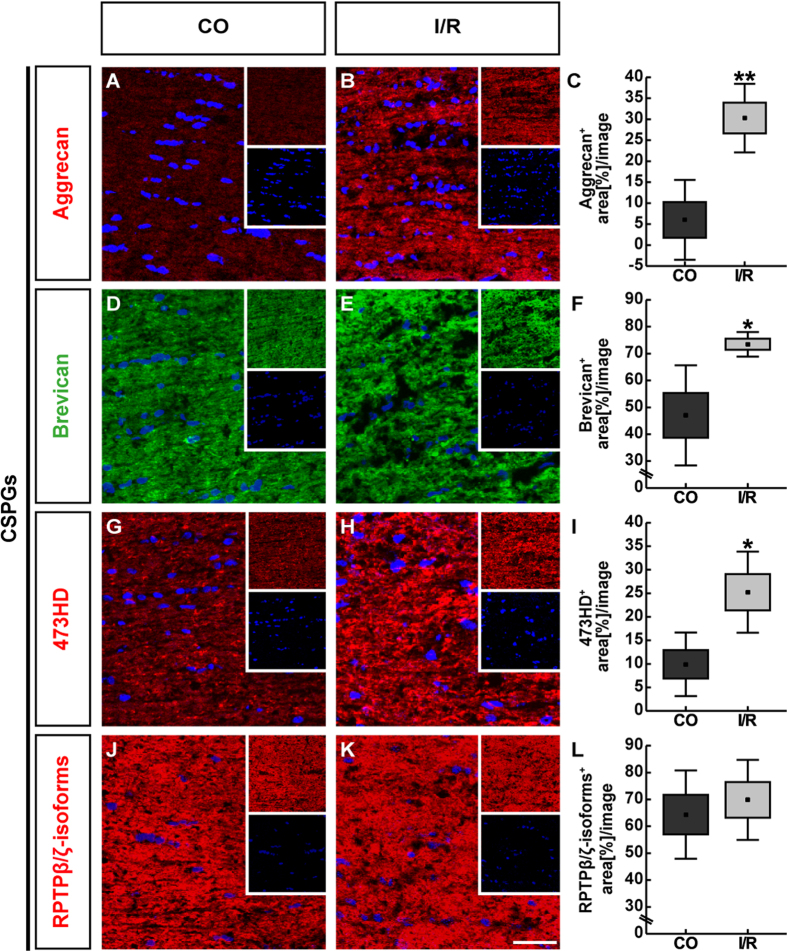
Representative longitudinal optic nerve sections of control (CO) and ischemic (I/R) eyes labelled using specific antibodies against the CSPGs aggrecan (**A**,**B**, red), brevican (**D**,**E**, green), phosphacan/RPTPβ/ζ_long_ (473HD antibody, **G**,**H**, red), RPTPβ/ζ-isoforms (**J**,**K**, red) as well as the nuclear dye TO-PRO-3 (blue) shown as merged images. Small inserts show TO-PRO-3 (blue) and CSPG (green/red) staining separately. The investigated CSPGs showed immunostaining throughout the optic nerve tissue. A dotted-like pattern was observed for 473HD immunostaining. Our evaluation revealed a significant increase in stained area for aggrecan, brevican and 473HD in ischemic optic nerves. No differences were found regarding phosphacan/RPTPβ/ζ staining area (**C**,**F**,**I**,**L**). Values are indicated as mean ± SEM ± SD. *p ≤ 0.05; **p ≤ 0.01; n = 5/group. Scale bar = 50 μm.

We also evaluated the mRNA and protein levels of the CSPGs aggrecan, brevican as well as of phosphacan/RPTPβ/ζ-isoforms in the optic nerves of CO and ischemic eyes (Figs 9A–D and 10A–L).

No significant difference in the *aggrecan* mRNA level was observed (0.84-fold, p = 0.378; Fig. 9A). On the other hand, extracellular aggrecan immunostaining was significantly increased in the I/R optic nerves (CO: 6.02 ± 9.50 area [%]/image; I/R: 30.28 ± 8.16 area [%]/image; p = 0.003; Fig. 10A–C).

A prominent and significant increase in mRNA expression levels of *brevican* (1.35-fold, p = 0.002) and *phosphacan/RPTPβ/ζ (RPTPβ/ζ CA:* 1.26-fold, p = 0.019; *RPTPβ/ζ PTP1:* 1.39-fold, p = 0.015) was found in optic nerves of the I/R group (Fig. 9B–D). Also, our immunohistochemical analyses revealed an increased planar staining pattern of brevican and 473HD in the ischemic optic nerve. Subsequent semi-quantitative analyses verified a significant increase in brevican (CO: 47.00 ± 18.65 area [%]/image; I/R: 73.48 ± 4.57 area [%]/image; p = 0.015; Fig. 10D–F) as well as in 473HD staining area (CO: 9.89 ± 6.75 area [%]/image; I/R: 25.24 ± 8.62 area [%]/image; p = 0.014; Fig. 10G–I). In contrast, phosphacan/RPTPβ/ζ staining area was comparable in optic nerves of both groups (CO: 64.37 ± 16.44 area [%]/image; I/R: 69.83 ± 14.88 area [%]/image; p = 0.597; Fig. 10J–L).

## Discussion

ECM remodeling upon retinal damage was reported by numerous previous studies. Nevertheless, little information is available regarding the significance of ECM remodeling following ischemic retinal injury. In the present study, we subsequently analyzed the expression and distribution pattern of several extracellular glycoproteins and CSPGs in the retina and optic nerve of an I/R rat model via qRT-PCR and immunohistochemistry. Additionally, retinal protein levels were quantified via Western blot analyses. Our results demonstrate that each ECM molecule displays an unique spatial expression and protein regulation, which reflects their potential functional role during ischemic degeneration.

### Dysregulation of ECM glycoproteins under ischemic conditions

We first monitored the expression pattern of the ECM glycoproteins fibronectin, laminin, tenascin-C and tenascin-R. For fibronectin, a significantly increased mRNA as well as protein level was noted in the ischemic retina. No regulation was seen in the optic nerve. As revealed by others and our study, prominent fibronectin staining is mainly restricted to retinal blood vessels of the inner retina^24^,^25^. ECM constituents display a central functional importance during vascular development and neovascularization^26^, which is associated with severe retinal ischemia. Abnormalities in the ECM of the retinal microvasculature are common, e.g. in diabetic retinopathies^27^. In patients with diabetic retinopathy, fibronectin was overexpressed in retinal microvessels. Here, it was speculated that increased fibronectin synthesis and deposition by microvascular cells may modify cell-matrix interaction with functional consequences relevant to retinal damage^28^. Due to these and our findings, we assume that fibronectin upregulation in the ischemic retina reflects substantial blood vessel remodeling, sprouting and/or neovascularization. The precise function of fibronectin during retinal angiogenesis is still largely unknown. It’s crucial importance in angiogenesis is underscored by knockout mice that exhibit severe vascular defects^29^. Stenzel *et al*. proposed that retinal astrocytes represent a major cellular source of fibronectin. Moreover, they provide evidence that its binding to vascular endothelial growth factor (VEGF) is important for retinal angiogenesis^30^. Under pathological conditions, astrocytes of the human glaucomatous optic nerve head display an enhanced fibronectin expression upon transforming growth factor-β2 (TGF-β2) treatment^31^. Laminin also represents a major component of retinal vascular basement membranes^32^. In addition, high levels of this glycoprotein are associated with the ILM and the GCL. Indeed, laminin plays a key role in RGC survival and reduced expression levels have been associated with glaucoma and optic nerve damage^33^,^34^,^35^. Our results indicate a downregulation of *α1-laminin* mRNA level in the ischemic retina, while a comparable regulation of laminin was observed on protein level. Retinal laminin degradation was previously reported in an I/R mouse model^36^. In this model, laminin-β1-integrin signaling and activation of the focal adhesion kinase were shown to be essential for the survival of RGCs. In contrast, matrix metalloproteinase-9 upregulation and consecutive laminin degradation lead to decreased levels of β1-integrin in RGCs and a reduced expression of the pro-survival factor Bcl-xL. Furthermore, agonists that maintain β1-integrin-activation can prevent RGC death. This was demonstrated in a mouse model of hypoxia-stimulated proliferative retinopathy. Here, a synthetic agonist peptide of the receptor acts protective against retinal ischemia by inhibiting hypoxia-induced neovascularization^37^. In conclusion, retinal downregulation of *α1-*laminin in our ischemic model is in accordance with previous findings. Nevertheless, we found no alteration on protein level, which might indicate a different regulation of various laminin-chains. Following ischemia, significantly increased endothelial staining was observed in the optic nerve, which seems to correspond to the observed enhanced laminin levels.

Our results revealed a significantly decreased mRNA expression for the glycoprotein *tenascin-C* in the I/R retina. Although no reduction was found for total tenascin-C protein levels, our Western blot analyses verified a significant downregulation of the 250 kDa tenascin-C band. In the retina, amacrine and horizontal cells are the cellular source of tenascin-C and it is enriched in the plexiform synaptic layers^38^. In general, tenascin-C is a main structural component of synaptic sites^39^,^40^. In response to ischemic injury, the retina shows signs of structural alterations and neuronal remodeling, also defined as injury-induced plasticity, possibly to preserve or regain some of its neuronal connections^41^. Due to our findings, tenascin-C dysregulation in the synaptic strata might indicate I/R-inflicted damage and synaptic reorganization. Moreover, its downregulation might also reflect the impact on amacrine cells, which was previously reported in this model^19^.

Remodeling of tenascin-C was also reported in other tissues following ischemia. For instance, it is dynamically expressed following hepatic or myocardial ischemia/reperfusion injury^42^,^43^,^44^. Dysregulation of this glycoprotein was also monitored after cerebral ischemia^45^,^46^.

In the I/R optic nerve, we could verify a significant increase of tenascin-C staining area. Due to these findings, we assume that optic nerve astrocytes, which represent a main source of this glycoprotein, respond to the ischemic damage^22^,^47^. With ongoing central nervous system development tenascin-C is progressively downregulated, although pronounced re-expression is monitored following neurodegeneration or injury^48^,^49^. Previous studies exploring the significance of this glycoprotein demonstrate a dysregulation following glaucomatous damage. In a rat glaucoma model of ocular hypertension, tenascin-C levels were enhanced in the optic nerve head^50^. Elevated levels were also associated with reactive astrocytes in optic nerve heads of primary open-angle glaucoma patients^51^. Here, it was speculated that tenascin-C might act protective to RGC axons by providing a barrier for blood-derived factors that may cause further tissue damage. In this context, it might have a neuroprotective role in ischemic optic nerve tissue. Regarding glaucomatous damage, we also reported an upregulation of tenascin-C in an intraocular pressure-independent autoimmune glaucoma model^52^.

Our analyses revealed a significant larger tenascin-R staining area in the ischemic retina. Via quantitative RT-PCR of mRNA levels no regulation was observed. Also, via Western blotting comparable levels of the total tenascin-R protein were found in both groups. But, in line with the immunohistochemical data, the larger (180 kDa) tenascin-R isoform was significantly upregulated. In contrast, the small (160 kDa) isoform was significantly downregulated following retinal ischemia. These findings strongly indicate an isoform-specific regulation of tenascin-R under ischemic conditions. Both isoforms can be distinguished via their number of fibronectin-type III repeats (8 or 9) and the tendency to form dimers and trimers, respectively, although their significance is not well understood yet^53^,^54^.

In the retina, tenascin-R is associated with unmyelinated fasciculated RGC axons, although horizontal cells are the major cellular source of its transcripts. Consistently, an enrichment of this extracellular protein is evident in the OPL^55^,^56^, its functional importance in horizontal cells is not known. Nevertheless, based on the enhanced staining of tenascin-R upon retinal ischemia, horizontal cells seem to react to retinal damage.

In general, tenascin-R represents a well-defined repellent, growth-inhibiting ECM component of optic nerve fibers in several species^57^,^58^,^59^,^60^. In this context, the epidermal growth factor family member CALEB, which is dynamically regulated after optic nerve lesion, represents a favorable interaction partner of tenascin-R during RGC axon regeneration^61^. In the optic nerve, tenascin-R is restricted to the myelinated part. Here, mainly oligodendrocytes contain its transcripts^55^. In addition, spots of increased labeling of this glycoprotein can be found in nodes of Ranvier. Nevertheless, since CO and I/R optic nerves had comparable levels of tenascin-R, we assume that tenascin-R has a minor functional importance in the ischemic optic nerve.

### Especially proteoglycans of the lectican family display enhanced expression levels in the ischemic optic nerve

Additionally, we focused on the dysregulation of specific proteoglycans in the I/R retina and optic nerve. Proteoglycans can bind to several other ECM molecules and cell surface receptors and play a pivotal role in CNS, including the retina^62^. Enhanced CSPG levels are associated with pathological conditions in the CNS and represent major constituents of the glial scar. Moreover, CSPGs exert growth-inhibitory effects on axonal regeneration^13^. Aberrant expression of proteoglycans was previously reported in numerous retinal diseases, such as retinitis pigmentosa, age-related macular degeneration and myopia^63^,^64^,^65^,^66^. Yet, little is known about their potential role in the ischemic retina and optic nerve. The upregulation of decorin in a rat ischemia/reperfusion model indicates its contribution to damage and repair processes in the injured retina^18^. Additionally, Inatani *et al*. reported an upregulation of the proteoglycan neurocan in the retina following transient ischemia^17^. In a nerve crush model, CSPGs inhibit optic nerve regeneration^67^. Nevertheless, no reports exist regarding the expression of the CSPGs aggrecan, brevican and phosphacan/RPTPβ/ζ in the ischemic retina and optic nerve. In the present study, we provide first evidence for a dysregulation of these CSPGs in a retinal ischemia animal model. Although the investigated CSPGs showed a minor dysregulation in the ischemic retina, a prominent upregulation was observed in the optic nerve, suggesting a re-expression of the investigated proteins following nerve degeneration.

In our study, a significantly increased aggrecan immunoreactivity was found in the ischemic optic nerve, while reduced protein levels were noted in the ischemic retina. Aggrecan expression was previously investigated in a dystrophic rat model^68^. Here, no retinal dysregulation was found in comparison to non-dystrophic rats. As our analyses revealed significantly reduced levels, we speculate that this CSPG is specifically involved in the ischemic retinal degeneration process or the reorganization of the retina.

Nagel and colleagues observed remodeling of aggrecan in individual neurons of a focal cerebral ischemia model, suggesting it plays a role in neuronal reorganization^69^. This might explain the observed upregulation of aggrecan in the ischemic optic nerve. As shown recently, aggrecan inhibits growth of axonal fibers in an optic nerve crush model *in vivo*^70^. Therefore, under ischemic conditions, aggrecan represents a favorable candidate that inhibits axonal regeneration.

Following retinal I/R, we noted enhanced brevican levels in the optic nerve. Within the myelinated axons of the optic nerve, this lectican family member co-localizes with the ECM molecules phosphacan and tenascin-R at the perinodal Ranvier nodes^71^,^72^. Regarding these findings and the observed upregulation of brevican following ischemia, we assume that ischemic damage might lead to a reorganization of the nodal matrix. A possible reorganization and associated functional consequences should be investigated in future studies. Our findings indicate a downregulation of *brevican* mRNA expression levels in the ischemic retina, although no regulation was observed on protein level. Specifically, reduced levels of brevican and proteolysis were described post hypoxic-ischemic brain injury in the hippocampal matrix^73^. A marked reduction of brevican occurs around a phase of progressive cell death and injury. In a neonatal hypoxic-ischemic injury model, a severe decrease of brevican was observed in the cortex and hippocampus shortly after injury. Markedly, elevated levels were found at later points in time localizing to degenerated cells within and in close association with the lesion core^74^. Interestingly, reduced brevican levels were also noted in the contralesional site of the striatum^75^.

Stronger phosphacan expression was reported in the degenerated optic nerve following partial transection^76^. In addition, we recently verified a transient upregulation of phosphacan in the optic nerve of an experimental autoimmune glaucoma model^52^. This is in agreement with our current findings, which reveal a significant upregulation of phosphacan/RPTPβ/ζ in the ischemic optic nerve. In the optic nerve, glial cells represent the cellular source of phosphacan/RPTPβ/ζ. The increased expression within the ischemic optic nerve might indicate that glia cells respond to this damage. Indeed, after acute ischemia/reperfusion activation of glia cells was reported^19^,^77^. Contrary to the observed upregulation of phosphacan/RPTPβ/ζ in the optic nerve, we noted a significantly reduced staining area in the ischemic retina. Here, we assume that retinal degeneration and accompanied gliosis precede optic nerve degeneration. In addition, Western blot analyses revealed a significantly decrease of the intermediate protein band after ischemia, which indicates a shift in the expression of RPTPβ/ζ isoforms, proteolytic products or the glycosylation pattern.

### Tenascin-C and phosphacan/RPTPβ/ζ show a corresponding expression pattern after ischemic damage

In nervous tissue phosphacan/RPTPβ/ζ represents a well characterized interaction partner of a variety of cell surface receptors, adhesion molecules, growth factors as well as ECM molecules, including tenascin-C^78^,^79^. Most importantly, our study revealed that phosphacan/RPTPβ/ζ and tenascin-C exhibit a corresponding expression profile in the investigated ischemic tissues. While tenascin-C and phosphacan/RPTPβ/ζ protein levels were differentially regulated in the ischemic retina, both proteins displayed a significantly increased immunoreactivity within the ischemic optic nerve. Concerning these findings, it is tempting to speculate that possibly divergent signaling of both interaction partners seems to depend on the tissue, cellular source as well as the level and time point of ischemic damage.

## Conclusion

In sum, we monitored a contribution of ECM remodeling in an I/R rat model. Our findings suggest that ECM glycoproteins and CSPGs display a unique expression profile and might play a role in ischemic retina and following optic nerve degeneration. Additional studies are necessary to delineate the functional processes underlying I/R injury. Still, our findings offer novel insights how ECM molecules contribute to ischemic damage.

## Methods

### Animals and ethics statement

Adult male brown Norway rats (7 weeks of age), purchased from Charles River Laboratories (Sulzfeld, Germany), were housed under a twelve-hour light-dark cycle with continuous access to food and water under pathogen-free conditions in the animal facility (Faculty of Medicine, Ruhr-University Bochum). All experiments were approved by the animal care committee of North Rhine-Westphalia, Germany, and were performed according to the ARVO statement for the use of animals in ophthalmic and vision research.

### Ischemia/reperfusion (I/R)

Ischemia/reperfusion was performed as described previously^19^,^80^. Briefly, animals were anesthetized using a mixture of ketamine (0.65 ml), xylazine (0.65 ml) and vetranquil (0.2 ml). The right eyes were dilated using 5% tropicamide (Pharma Stulln GmbH, Stulln, Germany) followed by topical anesthesia using conjuncain EDO (Bausch & Lomb GmbH, Berlin, Germany) and a subcutaneous injection of carprofen (0.1 ml/200 g; Pfizer Deutschland GmbH, Berlin, Germany) to block inflammation. Retinal ischemia was induced for 60 minutes by cannulation of the anterior chamber using a 27-gauge needle (Terumo Europe, Leuven, Belgium) connected to a reservoir containing 0.9% NaCl (Fresenius SE & Co. KGaA, Bad Homburg, Germany). Intraocular pressure (IOP) was raised to 140 mmHg for 60 min. Retinal ischemia was controlled via an ophthalmoscope (Mini 300; Heine Optotechnik, Herrsching, Germany). The left eyes served as untreated controls. Three weeks after I/R retina and optic nerve tissue was processed for quantitative real-time-PCR (retina and optic nerve: n = 4/group), immunohistochemistry (retina and optic nerve: n = 5/group) and Western blot analysis (retina: n = 5/group).

### RNA isolation, cDNA synthesis and quantitative real-time PCR analyses

For RNA preparation, retinal tissue from control and I/R rats was isolated twenty-one days after I/R, snap frozen in liquid nitrogen and stored in lysis buffer (Gene Elute Mammalian Total RNA Miniprep Kit; Sigma-Aldrich, Mannheim, Germany) at −80 °C until RNA extraction. Total RNA from each retina was extracted following the manufacturer’s instructions using the Gene Elute Mammalian Total RNA Miniprep Kit (Sigma-Aldrich, Mannheim, Germany). For total RNA isolation from optic nerve tissue, the ReliaPrep^TM^ RNA Tissue Miniprep System (Promega, Madison, USA) was used. RNA purity and concentration was quantified by spectrophotometry (BioSpectrometer, Eppendorf, Hamburg, Germany). To obtain cDNA, 1 μg of total RNA was reverse-transcripted by means of a cDNA-synthesis kit and random hexamer primers (Thermo Fisher Scientific, Waltham, MA, USA). Quantitative real-time PCR (qRT-PCR) experiments were performed with SYBR Green I in a Light Cycler 96 (Roche Applied Science, Mannheim, Germany). Primer efficiencies of each primer set (Table 1) were calculated based on a dilution series of 5, 25 and 125 ng cDNA. For normalization and relative quantification, the Ct values of the housekeeping genes *β-actin* (retina) and *cyclophilin* (optic nerve) were taken into account.

### Immunohistochemistry and confocal laser scanning microscopy

Eyes were enucleated, fixed in 4% paraformaldehyde (PFA), cryo-protected and embedded in Tissue-Tek freezing medium (Thermo Fisher Scientific, Cheshire, UK). Retinal tissue-sections (10 μm) were cut using a cryostat (Thermo Fisher Scientific, Walldorf, Germany) and collected onto Superfrost plus object slides (Menzel-Glaeser, Braunschweig, Germany). For immunohistochemistry, retinal cross-sections were dried and rehydrated. Cross-sections were blocked for 1 h at room temperature in blocking solution containing 1% normal goat or donkey serum (both Dianova, Hamburg, Germany), 1% w/v bovine serum albumin (BSA; Sigma-Aldrich) and 0.5% Triton-X-100 (Sigma-Aldrich) in PBS. All primary antibodies were diluted in blocking solution and were applied at room temperature overnight (Table 2). After washing in PBS, adequate secondary antibodies were applied and incubated at room temperature for 2 h. Fluorescent images (four images per two retinae; three images per three optic nerves) were captured by using a confocal laser-scanning microscope (LSM 510 META; Zeiss, Göttingen, Germany). Laser lines and emission filters were optimized using the Zeiss LSM Image Browser software. Staining signal areas were analyzed using ImageJ software (ImageJ 1.47t, National Institutes of Health, Bethesda, MD, USA) as described previously^52^,^81^. Briefly, photos were transferred into greyscale pictures. Then, background subtraction and upper and lower threshold were determined for each staining individually (Table 3). The percentage of each staining was determined and values were transferred to Statistica software (V 12; Statsoft, Tulsa, OK, USA) for statistical evaluation.

### SDS-PAGE and Western blotting

Control and ischemic retinal tissue was homogenized in 200 μl lysis buffer (60 mM n-octyl-β-D-glucopyranoside, 50 mM sodium acetate, 50 mM Tris chloride (pH 8.0) and 2 M urea) containing a protease inhibitor cocktail (Sigma-Aldrich). The protein homogenate was centrifuged at 14.000 x g at 4 °C for 30 min. Afterwards the supernatant was used for determination of protein concentration using a BCA Protein Assay kit (Pierce; Thermo Fisher Scientific, Rockford, IL, USA) following the manufacturer’s instructions. Next, 4x SDS sample buffer was added to each protein sample (20 μg). Then, samples were denaturized at 95 °C for 5 min and separated by SDS-PAGE (4–10% polyacrylamide gradient gels). Via Western blotting separated proteins were transferred to polyvinylidene difluoride (PVDF) membranes (Roth, Karlsruhe, Germany). Membranes were incubated in blocking solution (5% w/v milk powder in TRIS-buffered saline (TBS) and Tween 20, TBST) at room temperature for 1 h. Primary antibodies were diluted (Table 4) in blocking solution and applied on PVDF membranes overnight. After washing in TBST, appropriate horseradish peroxidase (HRP)- or biotin-coupled secondary antibodies (Table 4) were diluted in blocking solution and applied. Following incubation at room temperature for 1 h, membranes were washed. For protein detection, an ECL Substrate (Bio-Rad Laboratories GmbH, München, Germany) was mixed 1:1, added to the membranes and incubated for 5 min. Afterwards, protein immunoreactivity was documented using a MicroChemi Chemilumiscence Reader (Biostep, Burkhardtsdorf, Germany). Protein intensities were measured using ImageJ software. The intensity of the protein levels was normalized to the reference protein α-tubulin (Table 4). Here, each blot was re-probed.

### Statistical analyses

Immunohistochemical and Western blot data from control and I/R groups were analyzed by using the unpaired Student’s *t*-test and presented as mean ± standard error mean (SEM) ± standard deviation (SD). Data of qRT-PCR were presented as median ± quartile ± minimum ± maximum and analyzed by a pairwise fixed reallocation and randomization test (REST software). For all statistical analyses values of p ≤ 0.05 were considered significant.

## Additional Information

**How to cite this article**: Reinhard, J. *et al*. Ischemic injury leads to extracellular matrix alterations in retina and optic nerve. *Sci. Rep.* **7**, 43470; doi: 10.1038/srep43470 (2017).

**Publisher's note:** Springer Nature remains neutral with regard to jurisdictional claims in published maps and institutional affiliations.

## Figures and Tables

**Table 1:** List of primer pairs used for analyses of ECM glycoprotein and CSPG mRNA expression in control (CO) and ischemic (I/R) rat retinae and optic nerves by qRT-PCR.

Gene	Primer sequence	Amplicon size	Primer efficiency (retina/optic nerve)	Reference
*β-Actin_for*	CCCGCGAGTACAACCTTCT	72 bp	1.000/−	52
*β-Actin_rev*	CGTCATCCATGGCGAACT			
*Aggrecan_for*	TGGCTGCAGGACCAGACT	97 bp	1.000/1.000	This study
*Aggrecan_rev*	CGCCATAGGTCCTGACTCC			
*Brevican_for*	AGCAGAACCGCTTCAATGTC	61 bp	0.825/0.838	This study
*Brevican_rev*	TCAGAGAAGGCAGAGGGATG			
*Cyclophilin_for*	TGCTGGACCAAACACAAATG	88 bp	−/0.939	52
*Cyclophilin_rev*	CTTCCCAAAGACCACATGCT			
*Fibronectin_for*	CAGCCCCTGATTGGAGTC	73 bp	0.909/0.775	This study
*Fibronectin_rev*	TGGGTGACACCTGAGTGAAC			
*α1-Laminin_for*	CCCTGGGATGAAGAAGCA	101 bp	0.894/0.752	This study
*α1-Laminin_rev*	CAGGGCTGCTGATGGAAG			
*RPTPβ/ζ CA_for*	AACCATCCTTGGAAAACACG	66 bp	1.000/0.757	52
*RPTPβ/ζ CA_rev*	CATTGGTGAGATTTATTTCCACTGT			
*RPTPβ/ζ PTP1_for*	CCTCGTGGAGAAAGGAAGAAG	77 bp	0.953/0.739	52
*RPTPβ/ζ PTP1_rev*	CCAGGAAGCTCCCGTATTCT			
*Tenascin-C_for*	GCTCTCCTATGGCATCAAGG	60 bp	0.805/1.000	52
*Tenascin-C_rev*	TCATGTGTGAGGTCGATGGT			
*Tenascin-R_for*	TCATCTCCATTACTGCTGAGAGG	93 bp	0.769/0.779	This study
*Tenascin-R_rev*	AGTGCAAGTGGGAGATAGGG			

For relative quantification of mRNA levels in the retinae and optic nerves the genes *β-actin* and *cyclophilin* served as housekeeping genes, respectively. The primer sequence, the predicted amplicon size, primer efficiency for retinae and optic nerves and the reference are indicated. Abbreviations: bp = base pairs, for = forward, rev = reverse.

**Table 2:** List of primary antibodies and adequate secondary antibodies to analyze ECM glycoproteins and CSPGs in the retina and optic nerve of control and ischemic eyes via immunohistochemistry.

ECM proteins	Primary antibody	Dilution	Reference/source	Secondary antibody	Dilution	Source
Glycoproteins	Anti-fibronectin	1:300	82,83	Goat anti-rabbit Cy3	1:300	Dianova
	Anti-laminin	1:300	82,83	Goat anti-rabbit Cy2	1:300	Dianova
	Anti-tenascin-C (KAF14 antibody)	1:250	84	Goat anti-rabbit Cy2	1:250	Dianova
	Anti-tenascin-R (23–14 antibody)	1:100	85	Goat anti-mouse Cy3	1:250	Dianova
CSPGs	Anti-aggrecan	1:250	Merck Millipore	Goat anti-rabbit Cy3	1:300	Dianova
	Anti-brevican	1:300	86	Goat anti-guinea pig Cy2	1:300	Dianova
	Anti-phosphacan/ RPTPβ/ζ_long_ (473HD antibody)	1:200	20	Goat anti-rat Cy3	1:250	Dianova
	Anti-RPTPβ/ζ-isoforms (KAF13 antibody)	1:200	20	Goat anti-rabbit Cy3	1:250	Dianova

**Table 3:** Adjustments set for the ImageJ macro.

Glycoprotein/CSPG	Tissue	Background subtraction (pixel)	Lower threshold	Upper threshold
Aggrecan	Retina	50	16.21	85
	Optic nerve	50	14.13	85
Brevican	Retina	100	16.88	85
	Optic nerve	50	20.40	85
Fibronectin	Retina	50	16.39	85
	Optic nerve	50	15.07	85
Laminin	Retina	50	19.84	85
	Optic nerve	50	12.92	85
Phosphacan/RPTPβ/ζ_long_ (473HD antibody)	Retina	50	7.35	85
	Optic nerve	50	21.58	85
RPTPβ/ζ-isoforms (KAF13 antibody)	Retina	50	11.58	85
	Optic nerve	50	20.26	85
Tenascin-C (KAF14 antibody)	Retina	50	9.47	85
	Optic nerve	10	19.95	85
Tenascin-R	Retina	50	22.43	85
	Optic nerve	10	10.57	85

For area analyses of retinae and optic nerves, background subtraction as well as the lower and upper threshold was set for each stain as indicated.

**Table 4:** List of primary antibodies and appropriate secondary antibodies to analyze ECM glycoproteins and CSPGs in the retina of control and ischemic eyes via Western blotting.

ECM proteins	Primary antibody	Molecular weight	Dilution	Reference/source	Secondary antibody	Dilution	Source
Glycoproteins	Anti-fibronectin	>250 kDa	1:10.000	82,83	Goat anti-rabbit HRP	1:10.000	Dianova
	Anti-laminin	200 kDa, 400 kDa	1:10.000	82,83	Goat anti-rabbit HRP	1:5.000	Dianova
	Anti-tenascin-C (KAF14 antibody)	~250 kDa, >250 kDa	1:5.000	84	Goat anti-rabbit HRP	1:10.000	Dianova
	Anti-tenascin-R (23–14 antibody)	160 kDa, 180 KDa	1:1.000	85	Goat anti-mouse HRP	1:5.000	Dianova
CSPGs	Anti-aggrecan	>100 kDa, >150 KDa	1:1.000	Merck Millipore	Goat anti-rabbit HRP	1:10.000	Dianova
	Anti-brevican	~50 kDa, >100 kDa	1:1.000	86	Goat anti-guinea pig biotin	1:5.000	Dianova
	Anti-phosphacan/ RPTPβ/ζ_long_ (473HD antibody)	>150 kDa	1:100	20	Goat anti-rat HRP	1:5.000	Dianova
	Anti-RPTPβ/ζ-isoforms (KAF13 antibody)	>150 kDa	1:5.000	20	Goat anti-rabbit HRP	1:5.000	Dianova
House-keeping protein	Anti-α-tubulin (clone DM1A)	~50 kDa	1:20.000	Sigma-Aldrich	Goat anti-mouse HRP	1:10.000	Dianova

kDa = kilodalton.
